# Case report: A case report of Alport syndrome caused by a novel mutation of *COL4A5*

**DOI:** 10.3389/fgene.2023.1216809

**Published:** 2023-07-17

**Authors:** Shujun Pan, Rizhen Yu, Shikai Liang

**Affiliations:** ^1^ Clinical School of Medicine, Hangzhou Normal University, Hangzhou, Zhejiang, China; ^2^ Urology & Nephrology Center, Department of Nephrology, Zhejiang Provincial People’s Hospital, Affiliated People’s Hospital, Hangzhou Medical College, Hangzhou, Zhejiang, China

**Keywords:** Alport syndrome, *COL4A5*, case report, glycine substitution, genetic disease

## Abstract

Alport syndrome (#308940) is an X-linked genetic disease with clinical manifestations, such as hematuria, proteinuria, renal insufficiency, and end-stage renal disease. The disease is characterized by the thinning of the glomerular basement membrane in the early stages and the thickening of the glomerular basement membrane in the late stages and may be associated with ocular lesions and varying degrees of sensorineural deafness. Herein, we report a case of Alport syndrome caused by a *de novo* mutation in *COL4A5*. The patient was a young male with clinical manifestations of hematuria and massive proteinuria who was diagnosed with Alport syndrome based on renal pathology and genetic testing.

## 1 Introduction

The disease was first described by Arthur C. Alport in 1927 (Alport, 1927) and was named “Alport syndrome” (AS) in 1961. AS is a genetically and phenotypically heterogeneous disease affecting the glomeruli, cochlea, and ocular basement membrane caused by mutations in collagen IV genes, *COL4A3*, *COL4A4*, and *COL4A5* (Kashtan, 2021), which can lead to hematuria, proteinuria, and chronic progressive renal dysfunction. Some patients present with sensorineural hearing loss, retinopathy, or other extrarenal manifestations (Savige et al., 2015).

We report a case of Alport syndrome caused by a *de novo* mutation in *COL4A5*. This patient was encountered with a hemizygous mutation of *COL4A5* c.3604G>A, which is rare and helpful in improving clinicians’ understanding of Alport syndrome.

## 2 Case description

We report a case of a 27-year-old young man who underwent physical examination in our hospital, and the following values were revealed: urine protein ++++, urine occult blood +++, total protein 54.8 g/L, albumin 32.1 g/L, uric acid 538 μmol/L, total cholesterol 7.49 mmol/L, and triglycerides 2.29 mmol/L. Emission computed tomography (ECT) revealed mildly impaired filtration function and significantly delayed excretion, with a bilateral renal GFR of 75.01 mL/min. He experienced back pain and yellowing of urine after ECT. After admission, the patient’s laboratory test results showed the following values: urine protein +++, urine occult blood +++, albumin 1,480 mg/L, urine microalbumin/creatinine 117.7 mg/mmol, IgG 4.59 g/L, light chain κ 4.09 g/L, light chain λ 2.44 g/L, 24-h urinary protein 4,679.1 mg/24 h, total protein 49.9 g/L, albumin 31.1 g/L, globulin 18.8 g/L, uric acid 543 μmol/L, urea 8.86 mmol/L, and creatinine 96.5 μmol/L. No significant abnormalities were observed in the hearing test, and only myopia was observed upon eye examination.

**FIGURE 1:**
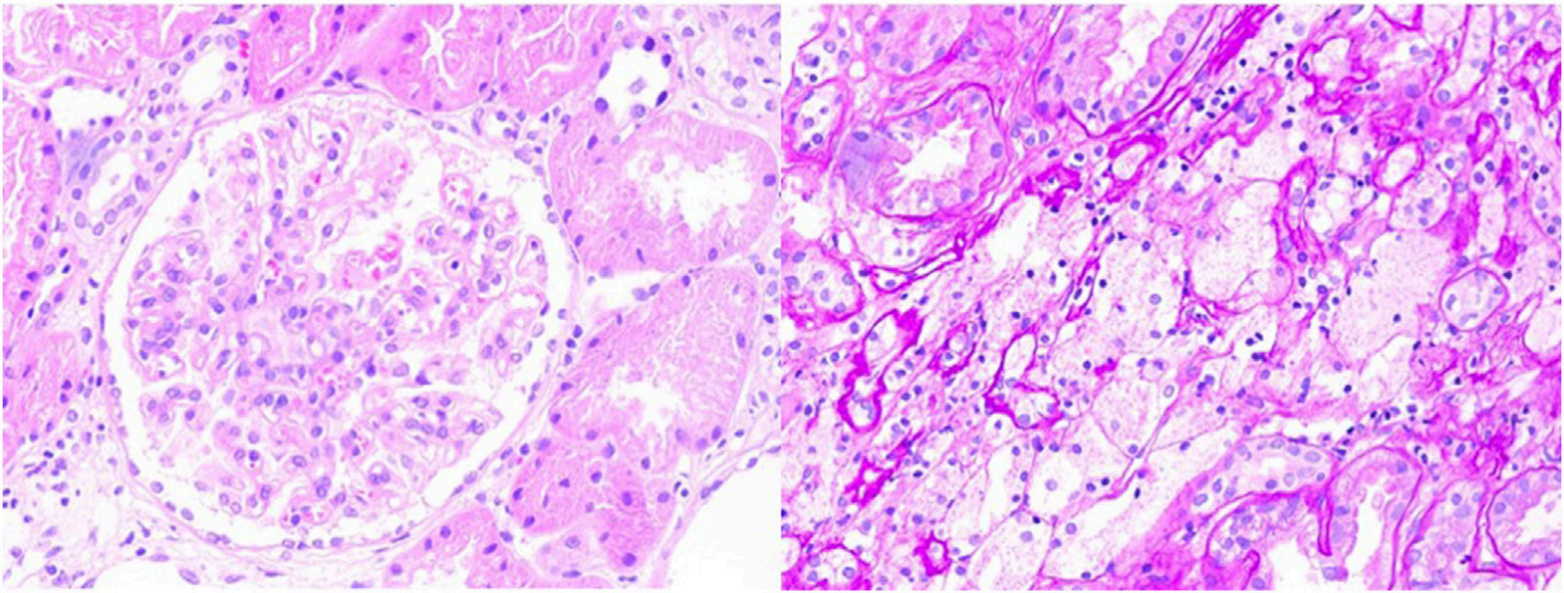
Light microscopic view of renal biopsy of the reported patient.

The initial diagnosis was nephrotic syndrome, and the patient underwent a renal biopsy to clarify the cause. As shown in Figure 1, light microscopy revealed two strips of cortical renal tissue, 12 glomeruli, one spherical sclerosis, one segmental sclerosis, one tubular glomerulus without glomeruli, mild widening of the remaining glomerular mesangial area, proliferation of mesangial cells, an increased mesangial matrix, well-developed capillary collaterals, and the segmental thickening of the Bowman’s capsule wall. PASM–Masson’s trichrome staining revealed equivocal complexophilic erythrocyte deposition and moderate chronic renal tubular interstitial lesions (approximately 25%) with multifocal tubular atrophy and basement membrane thickening. The multifocal tubular epithelial cells appeared turbid and swollen with fine granular degeneration; interstitial multifocal foam cell aggregates and infiltration of multifocal mononuclear, lymphatic, and plasma cells were observed. Immunofluorescence results showed negative for IgM, IgA, IgG, C3, C4, C1q, fibrin, HBs, HBc, IgG1, IgG2, IgG3, IgG4, κ, λ, and collagen IV α5 and positive for collagen IV α3 and collagen IV α1. Immunohistochemistry results showed negative for PLA2R. One glomerulus was detected through electron microscopy–light microscopy. Capillary endothelial cells were vacuolated and degenerated with erythrocytes visible in a single lumen; no obvious endothelial cell proliferation and open capillary loops were detected. No obvious thickening or stratification of the renal capsule wall layer or vacuolated degeneration of cells in the wall layer without notable hyperplasia was observed. The thickness of the basement membrane was variable, approximately 200–400 nm, and the dense layer of the basement membrane was thickened and partially torn and arachnoid-like. The epithelial cells in the visceral layer were swollen and vacuolated. Foot processes were mostly fused. The mesangial cells and stroma proliferated. No electron-dense deposits were observed. Vacuolar degeneration of renal tubular epithelial cells was observed. A few renal tubules were atrophic. The interstitium was infiltrated by a few inflammatory cells. Erythrocyte aggregates were observed in the lumen of individual capillaries. The walls of the small arteries were thickened.

The pathological presentation was first considered as Alport syndrome, and the patient was sent for genetic testing.

Genetic test results: the *COL4A5* c.3604G>A hemizygous mutation was detected in the peripheral blood DNA of the patient. According to the classification criteria of the American College of Medical Genetics and Genomics (ACMG) (Richards et al., 2015) for the clinical significance of genetic variants, c.3604G>A is a “potentially pathogenic variant.”

The patient was treated with diltiazem 30 mg twice daily, valsartan 80 mg once daily, and febuxostat 40 mg once daily. The patient was lost to follow-up.

## 3 Discussion and conclusion

AS is a hereditary renal disease with both monogenic and biallelic inheritances. More than 2,000 mutants of *COL4A5*, which encodes α3, α4, and α5 chains of collagen IV, have been identified, while approximately 500 mutants have also been described in *COL4A3* and *COL4A4*. Three classical Mendelian patterns of inheritance in AS exist: hemizygous X-linked due to mutations in *COL4A5* (XLAS; MIM#301050; 85% of patients), autosomal recessive due to mutations in the *COL4A3* or *COL4A4* gene (ARAS; MIM#203780; 10%–15% of patients), and autosomal dominant (ADAS; MIM#104200) (Kruegel et al., 2013; Tryggvason, 2021). Currently, a digenic inheritance (DI) of AS has been reported, which shows the occurrence of two mutations in the α3–4–5 collagen IV gene. Several studies (Fallerini et al., 2014; Fallerini et al., 2017) have identified double-gene inheritance in new AS families using next-generation sequencing, RNA studies, and clinical reassessments, revealing that digenic inheritance explains the highly variable clinical phenotype in AS better than single-gene inheritance. Although many of these are predicted to cause specific changes in the genes, the genotype–phenotype relationship is complex.

Autosomal recessive inheritance in female patients with AS leads to renal failure, hearing loss, keratoconus, and central retinopathy earlier than X-linked inheritance in female patients; however, affected relatives and the next generation of affected relatives are less likely to develop renal failure (Wang et al., 2014). Compared with X-linked and autosomal recessive inheritances in male patients, those with autosomal dominant inheritance have mild and slow renal manifestations, and extrarenal manifestations are relatively rare (Kamiyoshi et al., 2016; Furlano et al., 2021). Patients with double heterozygous mutations are believed to develop renal failure later than those with X-linked or autosomal recessive mutations. Despite similar genetic backgrounds, individual performance remains highly variable, and various modifier genes play important roles (Takemon et al., 2021). Mutants can be classified as in-frame, frameshift, missense, non-sense, and splicing mutations (Furlano et al., 2021). Severe mutations (large rearrangements, non-sense mutations, frameshift mutations, and splicing mutations) are associated with the earlier development of end-stage renal disease compared with missense mutations (Bekheirnia et al., 2010; Horinouchi et al., 2018; Mastrangelo et al., 2020).

AS is often diagnosed based on renal biopsy results. Genetic testing has become a non-invasive and definitive diagnostic technique (Adam et al., 2014; Braun et al., 2016; Mallett et al., 2017). In a 27-year-old man presented with hematuria and massive proteinuria, the histopathology of the kidney biopsy showed that the glomerular basement membrane was found with different thicknesses, and the dense layer of the basement membrane was thickened, some of which was torn and cobweb-like. Genetic testing revealed possible pathogenic missense mutations in *COL4A5*, which confirmed the diagnosis of AS.

**FIGURE 2:**
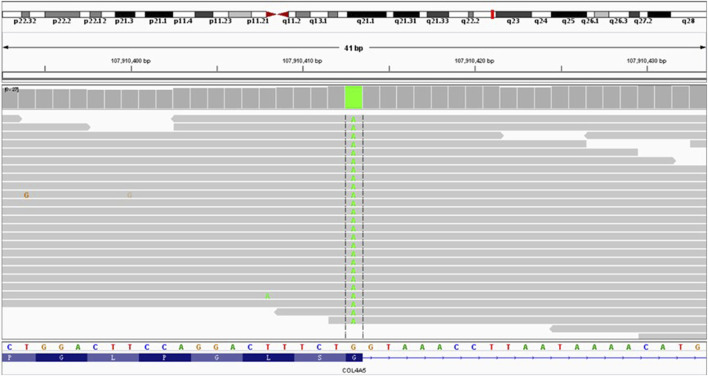
Results of *COL4A5* variant c.3604G>A sequencing and schematic representation of IGV.

**FIGURE 3:**
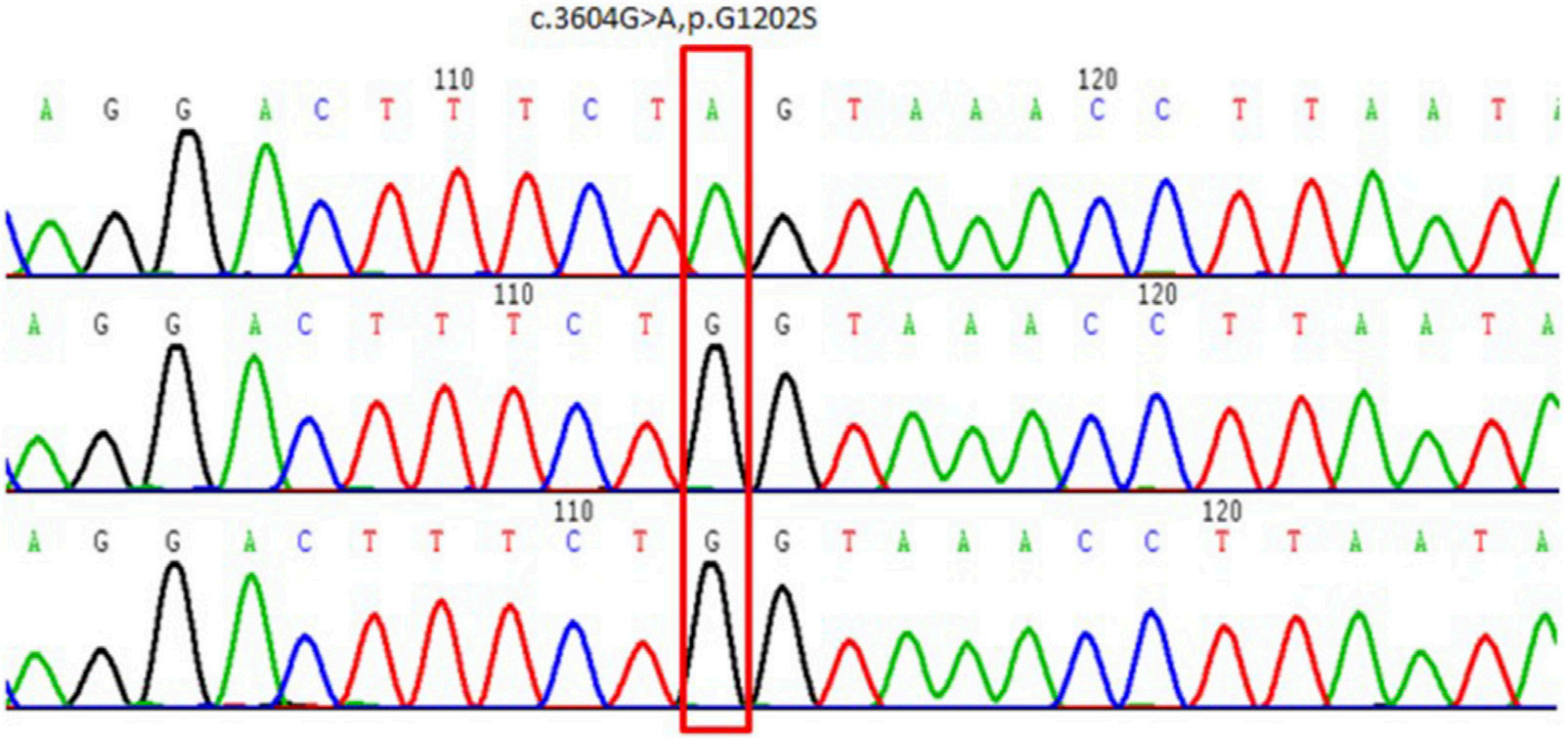
Schematic of the validation results by Sanger sequencing of *COL4A5* variants in the proband and his parents. Above: proband. Middle: proband’s mother. Below: proband’s father.

Whole-exome capture and sequencing of the genomic DNA of the patient and his parents revealed suspected pathogenic variants that could explain the patient’s phenotype. A mutation of base 3604 on the DNA of the coding region of *COL4A5* from G to A resulted in a mutation of amino acid 1202 in its coding region from glycine to serine (NM_000495.4:c.3604G>A, p.Gly1202Ser), as shown in Table 1. This variant has not been reported in the population database and is rare, with moderate evidence of pathogenicity (PM2). The variant is a novel missense mutation resulting in an amino acid change that has not been reported previously; however, a variant causing change in another amino acid at the same locus has been confirmed to be moderately pathogenic (PM5). This variant was not detected in the parental sequencing data and caused by a *de novo* mutation, providing strong pathogenic evidence (PS2). The results of the functional prediction software were biased toward pathogenic variants with a REVEL prediction of 0.872 and a CADD prediction of 25.2. This variant is a probable pathogenic variant according to the ACMG guidelines: 1PS + 2PM, which are shown in Figures 2, 3. The mutation was *de novo*, and neither parent harbored a mutation at this locus. The mode of inheritance was *de novo* X-linked.

**TABLE 1:** Gene mutation information of the patient.

Gene	NM No.	Nucleotide alteration	Amino acid change	Variant type	Homozygous/heterozygous	Source of variation	Genetic pattern	Clinical significance
*COL4A5*	NM_000495.4	c.3604G>A	p.Gly1202Ser	Missense	Hem	*De novo*	XLD	LP

Glycine substitutions are among the most common pathogenic variants in AS, disrupting Gly–X–Y triplets in the type IV collagen α5 chain (Yeo et al., 2020). Owing to the complex structure of the type IV collagen network, AS-associated glycine missense mutations can lead to the loss of function of a variety of proteins in the glomerular basement membrane (GBM) (Cosgrove and Liu, 2017). Glycine is the only amino acid that has no side chain substituents and can be bent to fill the triple helix structure, which is involved in the formation of disulfide bonds between *a*-chains essential for the formation of the triple helix structure. Alterations in GBM and abnormal interactions between stroma and podocytes can induce a pathological diagnosis of AS and lead to associated secondary pathological changes, which then cause proteinuria and secondary FSGS (focal segmental glomerulosclerosis) lesions (Savige and Harraka, 2021). This also explains the large amount of proteinuria and secondary FSGS lesions observed by light microscopy.

Single-base substitutions at the last nucleotide position in each exon affect the splicing pattern and may result in splice variants (Teraoka et al., 1999; Sahashi et al., 2007). However, in XLAS, these variants are generally considered missense if transcriptional analysis is not performed, which could potentially underestimate the phenotype of some patients. The patient in this case had a missense mutation in the end-exon nucleotide, which is likely to have affected the mRNA splicing process. Aoto et al. (2021) selected 20 mutations from the Human Gene Mutation Database (professional release 2021.1) and the study cohort, all of which were pathogenic variants caused by single-base substitutions in the last nucleotide position of exons (most of which were glutamate substitutions), and were subjected to splicing analysis. The results showed that 17 variants (85%) exhibited abnormal splicing, suggesting that a single-base substitution at the last nucleotide position in the *COL4A5* exon is likely to cause abnormal splicing. This study indicates that patients with aberrant splice variants exhibit a more severe renal prognosis than those with missense variants.

There is currently no specific treatment for AS. ACEI and ARB are often used to treat AS and delay renal failure (Yamamura et al., 2020b; Zhang et al., 2021; Boeckhaus et al., 2022; Zeng et al., 2023). Recently, many studies have demonstrated the protective effect of SGLT-2 inhibitors in kidney disease (Perkovic et al., 2019; Heerspink et al., 2020), and their therapeutic effects are still being investigated (Mabillard and Sayer, 2020). Hydroxypropyl-β-cyclodextrin (HPβCD) (Mitrofanova et al., 2018), 5-aryl nicotinamide compounds (Cpds) (Wright et al., 2021), and metformin (Omachi et al., 2021) showed positive therapeutic effects in a mouse model of AS. The development of exon-skipping therapy for X-linked AS with truncation variants of *COL4A5* (Yamamura et al., 2020a) has also shown positive effects.

In conclusion, patients with unexplained hematuria and proteinuria who do not respond to glucocorticoid therapy or who have extrarenal manifestations, such as chronic and progressive renal failure, hearing loss, and ocular abnormalities, should undergo renal biopsy and/or genetic testing as soon as possible to determine the etiology. The identification of *COL4A5* mutations in patients with suspected Alport syndrome confirms not only the diagnosis of X-linked disease but also the location and type of mutation, which help predict the clinical course and prognosis of the individual. Early diagnosis of AS is helpful in selecting a more appropriate treatment plan and assessing the patient prognosis. It also plays a guiding role for the patients’ family members in reproduction.

## Data Availability

The original contributions presented in the study are included in the article/Supplementary Materials, further inquiries can be directed to the corresponding author.

## Abbreviations

AS, Alport syndrome; ACMG, American Society of Medical Genetics and Genomics; ECT, emission computed tomography; HPβCD, hydroxypropyl-β-cyclodextrin; Cpds, 5-aryl nicotinamide compounds; GBM, glomerular basement membrane; FSGS, focal segmental glomerulosclerosis.
